# Distribution and Antibiotic Resistance of *Stenotrophomonas maltophilia* Isolates in a Tertiary Care Hospital in Türkiye: A Retrospective Study

**DOI:** 10.3390/antibiotics15070648

**Published:** 2026-06-30

**Authors:** Arzu Kayiş, Özlem Kirişçi, Zerife Orhan

**Affiliations:** 1 Medical Laboratory Techniques Program, Department of Medical Services and Techniques, Vocational School of Health Services, Kahramanmaras Sütçü İmam University, 46040 Kahramanmaras, Türkiye; zarifeorhan@ksu.edu.tr; 2 Department of Medical Microbiology, Faculty of Medicine, Kahramanmaras Sütçü İmam University, 46040 Kahramanmaras, Türkiye; dr_ozlemgitmisoglu@hotmail.com

**Keywords:** *Stenotrophomonas maltophilia*, antibiotic resistance, trimethoprim–sulfamethoxazole, prevalence, Türkiye

## Abstract

Objective: This study aimed to retrospectively analyze the demographic characteristics of patients, clinical specimen types, and antibiotic susceptibility patterns of *Stenotrophomonas maltophilia* (*S. maltophilia*) isolates obtained in a tertiary care hospital in Türkiye between 2019 and 2025. Materials and Methods: A total of 443 *S. maltophilia* strains isolated from various clinical specimens between June 2019 and December 2025 were included in the study. Identification of the isolates and antibiotic susceptibility testing were performed using conventional methods and an automated system. Demographic and clinical data of the patients, along with antibiotic susceptibility results, were obtained from the hospital information system. Statistical analyses were conducted using SPSS version 22.0 and R version 3.3.2. Results: The number of isolates showed a fluctuating trend over the years, with the highest number recorded in 2022 (n = 94). The majority of isolates were obtained from intensive care units (71.3%), followed by inpatient wards (21.9%). Among clinical specimens, blood (42.7%) and tracheal aspirates (18.5%) were the most common. Significant temporal changes were observed in TMP–SMX susceptibility patterns. Fully susceptible isolates predominated in the early years, whereas intermediate susceptibility became increasingly common after 2021, while resistance rates remained relatively low throughout the study period. Conclusion: *S. maltophilia* remains an important nosocomial pathogen, particularly in intensive care patients and in association with invasive procedures. The observed changes in susceptibility patterns may affect treatment efficacy. Therefore, regular resistance surveillance, and consideration of local antibiograms are of great importance.

## 1. Introduction

*Stenotrophomonas maltophilia* (*S. maltophilia*) is a facultative anaerobic, oxidase-negative, non-glucose-fermenting Gram-negative bacillus [1,2]. Before the 1970s, this bacterium was considered a rare opportunistic pathogen with low invasive potential; however, in parallel with advances in medical practice and the increasing number of immunosuppressed patients, it has been reported more frequently. Improvements in diagnostic methods, particularly the widespread use of automated identification systems and advanced technologies such as MALDI-TOF mass spectrometry, have enabled more accurate and rapid identification of this organism, leading to its recognition as an important nosocomial pathogen over time [3]. In recent years, the increasing frequency of both hospital-acquired and community-acquired infections has highlighted *S. maltophilia* as a significant multidrug-resistant pathogen. This organism, which is widely distributed in the environment, is particularly associated with severe infections in immunosuppressed patients, individuals in intensive care units, and those exposed to broad-spectrum antibiotics [4]. *S. maltophilia* can cause a wide range of infections, including ventilator-associated nosocomial pneumonia, catheter-related bloodstream infections, skin and soft tissue infections, endocarditis, meningitis, endophthalmitis, and urinary tract infections. Important risk factors for these infections include central venous catheterization and urinary catheter use, prolonged hospitalization, surgical interventions, intensive care stay, organ transplantation, neutropenia, empirical use of broad-spectrum antibiotics, cystic fibrosis, underlying malignancy, and HIV infection [1,5].

The treatment of *S. maltophilia* infections presents significant clinical challenges. The distinction between colonization and true invasive infection is not always clear, complicating clinical management. Additionally, the failure to recognize associated risk factors and clinical findings at an early stage may delay appropriate antibiotic therapy, thereby adversely affecting mortality [6]. Indeed, mortality rates associated with systemic infections caused by this microorganism have been reported to reach up to 69% [2].

*S. maltophilia* is intrinsically resistant to aminoglycosides and carbapenems due to its inherent resistance mechanisms [2,3,5]. This limits treatment options, and trimethoprim–sulfamethoxazole (TMP–SMX) is recommended as the first-line agent due to its high efficacy and favorable clinical outcomes [3,5,7]. However, its use may be limited by allergy, intolerance, and emerging resistance [3]. Studies conducted in different geographical regions have reported that tetracycline group antibiotics, such as minocycline, tigecycline, and doxycycline, may also be effective against *S. maltophilia* infections [8,9]. Furthermore, multidrug resistance and a strong biofilm-forming capacity make infections caused by this microorganism difficult to treat and increasingly important among healthcare-associated infections [10].

The aim of this study was to retrospectively analyze the demographic characteristics of patients, the types of clinical specimens, and the antibiotic resistance patterns of *S. maltophilia* isolates obtained from various clinical samples between June 2019 and December 2025 in a tertiary care hospital in Türkiye, and to present epidemiological and clinical data related to this pathogen.

## 2. Results

Of the total 443 *S. maltophilia* isolates obtained between 2019 and 2025, 286 (64.6%) were isolated from male patients and 157 (35.4%) from female patients.

**Figure 1:**
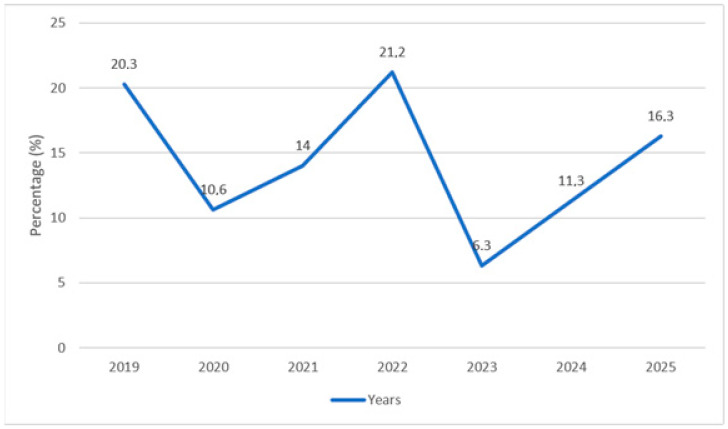
Annual percentage distribution of S. maltophilia isolates between 2019 and 2025.

It was determined that the number of *S. maltophilia* isolates obtained from various clinical specimens showed a fluctuating distribution over the years. The highest number of isolates was observed in 2022 (94/443, 21.2%), followed by 2019 (90/443, 20.3%) and 2025 (72/443, 16.3%). The lowest number of isolates was recorded in 2023 (28/443, 6.3%) (Figure 1).

**Figure 2:**
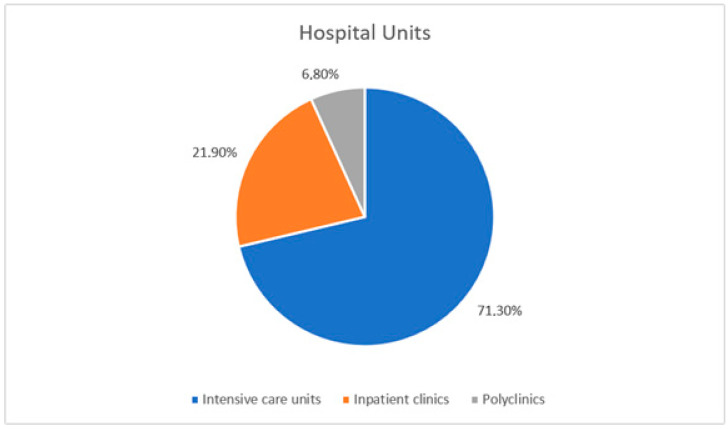
Distribution of *S. maltophilia* according to hospital units.

*S. maltophilia* was most frequently isolated from intensive care units (ICU) (71.3%), followed by inpatient wards (21.9%) (Figure 2).

When the annual distribution of *S. maltophilia* isolates according to hospital units was examined, it was observed that the isolates were most frequently obtained from intensive care units. Notably, in 2022, the proportion of ICU-derived isolates reached the highest level (25.3%). In contrast, the proportions of isolates obtained from inpatient wards and outpatient clinics were lower and more variable over the years. While ward-derived isolates showed a marked increase in 2024 and 2025, outpatient clinic-derived isolates generally remained at low levels.

When the distribution by years was compared, a statistically significant difference was found in the distribution of isolates across hospital units (χ^2^ = 37.108, *p* = 0.0001). This finding suggests that *S. maltophilia* infections are more frequently observed in intensive care units and that their clinical distribution varies over time (Table 1).

**Figure 3:**
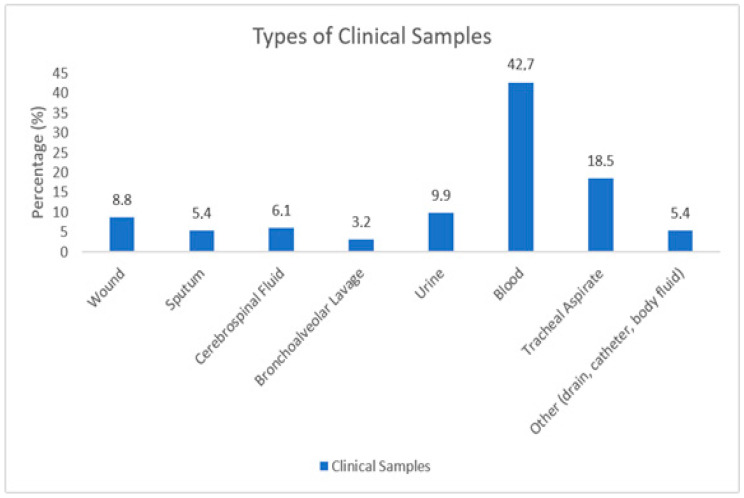
Distribution of *S. maltophilia* according to specimen types.

*S. maltophilia* was most frequently isolated from blood samples (42.7%) (only three blood culture samples were obtained through central venous catheters), followed by tracheal aspirates (18.5%), urine (9.9%), and wound samples (8.8%). In total, 326 (73.6%) isolates were identified as hospital-acquired infections (Figure 3).

When the distribution of *S. maltophilia* isolates over the years according to clinical specimen types was examined, marked differences were observed among specimen types. Notably, isolates obtained from blood samples were particularly high in 2022 and 2025. While a high proportion was observed in CSF samples in 2019, a significant decrease was noted in the following years. In contrast, an increasing trend over the years was observed in urine and tracheal aspirate samples.

The distribution of isolates from wound, sputum, and other specimen types appeared to be more variable. Overall, the distribution of isolates according to clinical specimen types showed a statistically significant difference between years (χ^2^ = 145.407, *p* < 0.001). This finding indicates that the frequency of *S. maltophilia* in different clinical specimens has changed over time, with periodic increases observed in certain specimen types (Table 2).

When the TMP–SMX susceptibility distribution of *S. maltophilia* isolates was examined, susceptible isolates were predominant in 2019 and 2020, whereas intermediate isolates were observed at the highest rate in 2021. From 2022 onward, intermediate isolates became clearly dominant. No susceptible isolates were detected in 2023, 2024, and 2025; in these years, nearly all isolates were classified as intermediate, with a small proportion of resistant isolates also observed. The distribution across years was found to be statistically significant (χ^2^ = 461.407, *p* < 0.001) (Table 3).

## 3. Discussion

*S. maltophilia*, an opportunistic pathogen, has attracted attention in recent years as an important healthcare-associated agent reported with increasing frequency [5]. In our study, 326 (73.6%) of the isolates were identified as hospital-acquired infections, further highlighting the clinical importance of this pathogen. In this study, the number of isolates obtained between 2019 and 2025 showed a fluctuating trend over the years (Figure 1). Similarly, a study by Kazak et al. [11] reported increases and decreases in isolate numbers during certain periods. Şen et al. [12] reported an overall increasing trend over the years, suggesting that lower isolation rates in some years might be related to the scope of the data.

The observed variability in isolate numbers may be attributed to annual differences in the intensive care unit patient profile and hospitalization rates, variations in the frequency of invasive procedures and mechanical ventilation use, changes in the use of broad-spectrum antibiotics, and periodic differences in the effectiveness of infection control measures.

In the present study, *S. maltophilia* was observed to be isolated at a higher rate in male patients. This finding is consistent with several studies reported in the literature [1,2,5,7,13,14,15]. The higher isolation rates observed in males are considered to be associated more with clinical and epidemiological factors rather than a gender-specific biological predisposition.

One of the important characteristics of *S. maltophilia* is its ability to form biofilms at the site of infection and to colonize both biotic and abiotic surfaces. This feature becomes more pronounced, particularly in conditions where the host immune system is weakened [16,17]. Invasive procedures such as mechanical ventilation, central venous catheterization, and urinary catheter use, along with intensive care unit stay, prolonged hospitalization, exposure to broad-spectrum antibiotics, and immunosuppression, are among the main risk factors for the development of infection. In addition, underlying malignancy, the presence of invasive devices, and chronic respiratory diseases such as cystic fibrosis are also reported as predisposing factors [3,13].

In our study, 71.3% of *S. maltophilia* isolates were obtained from patients hospitalized in intensive care units. This finding is consistent with studies in the literature reporting higher isolation rates among ICU patients [14,18]. The cumulative effect of existing risk factors in ICU patients likely contributes to the higher frequency of *S. maltophilia* infections in these units.

In this study, when hospital units were compared on a yearly basis, it was observed that the isolation rates of *S. maltophilia*, which were higher in intensive care units (Figure 2) during the early years (2019–2022), increased in inpatient wards in the later years (2023–2025) (Table 1). This finding suggests that infections were initially more ICU-associated but may have spread to other hospital units over time.

Although *S. maltophilia* is generally a microorganism that may be associated with colonization or contamination, it can also act as a causative agent of various clinical conditions, particularly lower respiratory tract and bloodstream infections [11]. In many studies in the literature, this microorganism has been reported to be most frequently isolated from respiratory tract specimens, followed by blood samples [1,4,12,13,18,19]. In our study, however, unlike some reports in the literature, *S. maltophilia* was most frequently isolated from blood (42.7%), followed by tracheal aspirates (18.5%) (Figure 3). Nevertheless, some studies have also reported high isolation rates from blood samples, and our findings are partially consistent with the literature in this respect [5,7,11]. The high rate of isolation from blood samples may be related to the patient profile in intensive care units, the frequency of invasive procedures, and the widespread use of central venous catheters. In addition, differences in patient populations and sampling practices between centers may also contribute to this finding.

The opportunistic nature of *S. maltophilia*, its association with intravascular catheters, its biofilm-forming capacity, and its selective advantage in immunosuppressed patient groups where broad-spectrum antibiotic use is common are among the possible factors explaining its more frequent isolation in bloodstream infections.

In our study, the distribution of *S. maltophilia* isolates according to clinical specimen types showed marked variability over the years. Notably, CSF samples were prominent in 2019, whereas blood cultures were more prominent in certain other periods. This finding indicates that *S. maltophilia* is an opportunistic pathogen that can present with different clinical manifestations and is sensitive to hospital-related dynamics. The increase in isolates obtained from blood cultures in some periods suggests that this organism may play an important role in bacteremia and catheter-related infections. This may be particularly associated with the high frequency of invasive procedures and prolonged hospitalization in intensive care unit patients. On the other hand, the increasing trend observed in urine and tracheal aspirate samples may reflect the impact of invasive practices such as urinary catheterization and mechanical ventilation.

This year-to-year variability may be related to differences in patient profiles and reasons for hospitalization, case distribution in intensive care units, the frequency of invasive procedures, and temporal changes in infection control practices. In addition, variations in clinical sampling approaches and a focus on specific patient groups may also contribute to these fluctuations. These findings highlight the need for continuous surveillance of different clinical specimen types and the strengthening of measures aimed at controlling invasive procedures.

The treatment of *S. maltophilia* infections is challenging due to the microorganism’s intrinsic and acquired resistance mechanisms against various antimicrobial agents, which limit available therapeutic options [18]. TMP/SMX has long been considered the first-line agent for the treatment of these infections [2]. Previous studies have reported high susceptibility of *S. maltophilia* clinical isolates to TMP/SMX, with some series indicating susceptibility rates as high as 100% [20].

However, in subsequent years, parallel to the increase in isolate numbers, a notable rise in resistance rates to various antibiotics, including TMP/SMX, has been reported. Supporting this observation, a study conducted in Malawi reported multidrug-resistant *S. maltophilia* isolates [21]. A recent meta-analysis demonstrated that the global resistance rate to TMP/SMX is approximately 9%, reaching higher levels of 19.2% in Asia [22]. Similarly, studies from China have reported a significant increase in TMP/SMX resistance over time, rising from approximately 19.2% in 2005 to 46.9% in 2014 [23]. Another study reported that resistance rates increased significantly from 29.7% in 2005–2009 to 47.1% in 2010–2014 [24]. In contrast, a study conducted in Mexico reported that all *S. maltophilia* isolates were resistant to TMP/SMX [25].

TMP/SMX resistance in *S. maltophilia* is associated with genetic mechanisms such as sul genes, integrons, and efflux pumps. In addition, the selective pressure resulting from the widespread use of broad-spectrum antibiotics contributes to the emergence of resistant strains. The intrinsic resistance of *S. maltophilia* to agents such as carbapenems provides a selective advantage under antibiotic pressure, facilitating its persistence and spread in hospital environments [6,8]. Furthermore, the higher frequency of *S. maltophilia* infections in healthcare-associated settings and in patient groups with high antibiotic exposure is considered an important factor facilitating the selection of resistant isolates in these environments [6].

Studies reported from our country indicate that TMP/SMX resistance rates of *S. maltophilia* may vary considerably between centers. While the resistance rate was reported as 1% in a study conducted in İzmir [12], it was reported as 1.3% in Konya [14]. A resistance rate of 2.7% was reported in Gaziantep [26], whereas in a study conducted in Van, this rate reached as high as 20.3% [27]. In a study from Diyarbakır, the susceptibility rate was reported as 57%, indicating approximately 43% resistance or reduced susceptibility [28]. These differences may be attributed to variations in antibiotic usage, testing methods, and patient populations.

Changes in TMP–SMX susceptibility category distribution were observed over the study period. However, because susceptibility interpretations were based on the EUCAST criteria applicable at the time of testing, part of the observed variation may be attributable to changes in interpretive criteria and breakpoint definitions rather than representing a true increase in resistance. The finding that all isolates were susceptible in 2019 reflects high susceptibility rates in the early period. However, starting from 2020, a decline in susceptibility was observed, which became more pronounced in 2021, when only 37.1% of isolates were susceptible, 54.84% were intermediate, and 8.06% were resistant. This year stands out as the period in which the most notable shift in the susceptibility profile occurred. From 2022 onward, the proportion of susceptible isolates decreased markedly, and the majority of isolates shifted to the intermediate category. Between 2023 and 2025, no susceptible isolates were detected; most isolates were classified as intermediate, while resistance rates remained at low levels (Table 3).

Overall, a shift in susceptibility category distribution was observed during the study period. However, because susceptibility interpretations were based on the EUCAST criteria applicable at the time of testing, these findings should not be interpreted as definitive evidence of increasing resistance over time. while the development of high-level resistance remained limited. These findings indicate a change in susceptibility category distribution over time; however, the contribution of evolving interpretive criteria should be considered when evaluating temporal trends. From a clinical perspective, this trend indicates an increasing proportion of borderline susceptibility, which may affect treatment success and highlights the importance of considering up-to-date susceptibility data in empirical therapy selection.

### Limitations

This study has several limitations. First, its retrospective and single-center design may limit the generalizability of the findings to other institutions and populations. Second, clinical data such as comorbidities, severity of illness, prior antibiotic exposure, and patient outcomes could not be evaluated in detail, which may have influenced the interpretation of the results. Third, the distinction between colonization and true infection could not always be clearly established, particularly for respiratory and catheter-related specimens.

In addition, antibiotic susceptibility testing was performed using an automated system, and confirmatory methods or molecular analyses to detect resistance mechanisms were not conducted. Therefore, the underlying genetic determinants of resistance, such as specific resistance genes or efflux mechanisms, could not be investigated. Finally, only TMP/SMX susceptibility was evaluated. Antibiotic sensitivities other than TMP/SMX were not examined. Therefore, data on alternative treatment options remain limited.

In addition, changes in EUCAST interpretive criteria during the study period may have influenced susceptibility category classifications and should be considered when interpreting temporal trends.

## 4. Materials and Methods

A total of 443 *S. maltophilia* isolates obtained from various clinical specimens sent to the Medical Microbiology Laboratory of Kahramanmaras Sütçü İmam University Health Practice and Research Hospital between June 2019 and December 2025 were included in this study. If growth was detected in multiple specimens from the same patient, only the first isolate was included in the analysis. Blood culture samples were incubated for 5 days using the BACT/ALERT 3D automated system (BioMérieux, Marcy l’Etoile, France). Bottles showing growth, as well as other specimens such as wound, sputum, cerebrospinal fluid (CSF), bronchoalveolar lavage (BAL), urine, and tracheal aspirates, were inoculated onto Eosin Methylene Blue (EMB) agar and blood agar media, and the plates were incubated at 37 °C for 24–48 h.

After incubation, isolates exhibiting typical colony morphology and characterized as oxidase-, urease-, and indole-negative, and capable of oxidizing glucose and maltose, were presumptively identified as *S. maltophilia*. This preliminary identification was confirmed using the Phoenix automated system (Becton Dickinson, Sparks, MD, USA). Antibiotic susceptibility testing of the isolates was performed using the Phoenix automated system. TMP–SMX susceptibility results were interpreted according to the EUCAST breakpoint tables routinely applied in our laboratory at the time of isolate testing. During the study period, antimicrobial susceptibility testing panels were prepared by prioritizing bacterial–antibiotic combinations for which EUCAST clinical breakpoints were available [29]. Therefore, routine susceptibility interpretation for S. maltophilia was limited to TMP–SMX. Demographic and clinical data of the patients (year, age, sex, specimen type, clinical department from which the specimen was obtained, and antibiogram results) were retrieved from the hospital laboratory information management system.

### Statistical Analysis

Data were analyzed using IBM SPSS version 22 (IBM SPSS for Windows, version 22, IBM Corporation, Armonk, NY, USA) and R version 3.3.2. Data were presented as numbers (n) and percentages (%). Differences between the frequency distributions of categorical variables were analyzed using the Chi-square test and Fisher’s exact test. A *p*-value of <0.05 was considered statistically significant.

## 5. Conclusions

In this study, the demographic distribution, clinical sources, and antibiotic susceptibility patterns of *S. maltophilia* isolates obtained in a tertiary care hospital between 2019 and 2025 were evaluated. The fact that most isolates were obtained from male patients and intensive care units indicates that this microorganism is an important opportunistic pathogen, particularly in high-risk patient groups.

A significant change in TMP–SMX susceptibility category distribution was observed over the study period. However, these findings should be interpreted in the context of the EUCAST criteria applied at the time of testing. This trend may have important implications for treatment success.

In conclusion, *S. maltophilia* remains an important nosocomial pathogen with a changing susceptibility profile, highlighting the importance of regular surveillance, treatment approaches based on local antibiograms, and rational antibiotic use.

## Figures and Tables

**Table 1:** Annual distribution of *S. maltophilia* isolates according to hospital units.

Years	Hospital Units			
	Intensive Care Units n (%)	Inpatient Clinics n (%)	Polyclinics n (%)	*p*-Value
2019	68 (21.5)	16 (16.5)	6 (20)	0.0001 * (χ^2^ = 37.108)
2020	38 (12)	9 (9.3)	0 (0.0)	
2021	45 (14.2)	13 (13.4)	4 (13.3)	
2022	80 (25.3)	8 (8.2)	6 (20)	
2023	16 (5.1)	8 (8.2)	4 (13.3)	
2024	28 (8.9)	19 (19.6)	3 (10)	
2025	41 (13)	24 (24.7)	7 (23.3)	

* *p* < 0.05 statistically significant; χ^2^: Chi-Square test.

**Table 2:** Annual distribution of clinical specimen types from which *S. maltophilia* was isolated.

Years	Clinical Samples								
	Wound n (%)	Sputum n (%)	CSF n (%)	BAL n (%)	Urine n (%)	Blood n (%)	Tracheal Aspirate n (%)	Other n (%)	*p*-Value
2019	8 (20.5)	5 (20.8)	20 (74.1)	3 (21.4)	3 (6.8)	25 (13.2)	21 (25.6)	5 (20.8)	0.0001 * (χ^2^ = 145.407)
2020	2 (5.1)	4 (16.7)	0 (0)	1 (7.1)	3 (6.8)	21 (11.1)	13 (15.9)	3 (12.5)	
2021	7 (17.9)	4 (16.7)	1 (3.7)	2 (14.3)	5 (11.4)	25 (13.2)	13 (15.9)	5 (20.8)	
2022	1 (2.6)	4 (16.7)	1 (3.7)	2 (14.3)	7 (15.9)	66 (34.9)	12 (14.6)	1 (4.2)	
2023	7 (17.9)	1 (4.2)	5 (18.5)	0 (0.0)	4 (9.1)	4 (2.1)	4 (4.9)	3 (12.5)	
2024	9 (23.1)	2 (8.3)	0 (0.0)	3 (21.4)	9 (20.5)	16 (8.5)	5 (6.1)	6 (25)	
2025	5 (12.8)	4 (16.7)	0 (0.0)	3 (21.4)	13 (29.5)	32 (16.9)	14 (17.1)	1 (4.2)	

* *p* < 0.05 statistically significant; χ^2^: Chi-Square test; CSF: cerebrospinal fluid, BAL: bronchoalveolar lavage, Other (drain, catheter, body fluid).

**Table 3:** Distribution of TMP–SMX susceptibility of *S. maltophilia* isolates by years.

Years	Sensitive n (%)	Moderately Sensitive n (%)	Resistance n (%)	Total	*p*-Value
2019	90 (100.00)	0 (0.00)	0 (0.00)	90	0.0001 * (χ^2^ = 461.407)
2020	45 (95.74)	0 (0.00)	2 (4.26)	47	
2021	23 (37.10)	34 (54.84)	5 (8.06)	62	
2022	6 (6.38)	88 (93.62)	0 (0.00)	94	
2023	0 (0.00)	27 (96.43)	1 (3.57)	28	
2024	0 (0.00)	50 (100.00)	0 (0.00)	50	
2025	0 (0.00)	71 (98.61)	1 (1.39)	72	
Total				443	

χ^2^: Chi-Square test. * Statistically significant (*p* < 0.05).

## Data Availability

The original contributions presented in this study are included in the article. For more information, please contact the corresponding author.

## Abbreviations

EUCAST	European Committee on Antimicrobial Susceptibility Testing
TMP–SMX	Trimethoprim–sulfamethoxazole
ICU	Intensive care units
CSF	Cerebrospinal fluid
BAL	Bronchoalveolar lavage
